# Interdomain flexibility and interfacial integrity of β-lactamase inhibitory protein (BLIP) modulate its binding to class A β-lactamases

**DOI:** 10.1016/j.jbc.2021.100980

**Published:** 2021-07-21

**Authors:** Liwen Huang, Pui-Kin So, Yu Wai Chen, Yun-Chung Leung, Zhong-Ping Yao

**Affiliations:** 1 State Key Laboratory of Chemical Biology and Drug Discovery, Research Institute for Future Food and Department of Applied Biology and Chemical Technology, The Hong Kong Polytechnic University, Hung Hom, Kowloon, Hong Kong Special Administrative Region, China; 2 State Key Laboratory of Chinese Medicine and Molecular Pharmacology (Incubation) and Shenzhen Key Laboratory of Food Biological Safety Control, Shenzhen Research Institute of The Hong Kong Polytechnic University, Shenzhen, China

**Keywords:** β-lactamases, β-lactamase inhibitory protein (BLIP), interdomain flexibility, interfacial integrity, hydrogen deuterium exchange mass spectrometry, molecular dynamics simulation, BLIP, β-lactamase inhibitory protein, CTD, C-terminal domain, HDX-MS, hydrogen deuterium exchange mass spectrometry, MD, molecular dynamics, RMSD, root-mean-square-deviation, SHV, sulfhydryl variant

## Abstract

β-Lactamase inhibitory protein (BLIP) consists of a tandem repeat of αβ domains conjugated by an interdomain loop and can effectively bind and inactivate class A β-lactamases, which are responsible for resistance of bacteria to β-lactam antibiotics. The varied ability of BLIP to bind different β-lactamases and the structural determinants for significant enhancement of BLIP variants with a point mutation are poorly understood. Here, we investigated the conformational dynamics of BLIP upon binding to three clinically prevalent class A β-lactamases (TEM1, SHV1, and PC1) with dissociation constants between subnanomolar and micromolar. Hydrogen deuterium exchange mass spectrometry revealed that the flexibility of the interdomain region was significantly suppressed upon strong binding to TEM1, but was not significantly changed upon weak binding to SHV1 or PC1. E73M and K74G mutations in the interdomain region improved binding affinity toward SHV1 and PC1, respectively, showing significantly increased flexibility of the interdomain region compared to the wild-type and favorable conformational changes upon binding. In contrast, more rigidity of the interfacial loop 135–145 was observed in these BLIP mutants in both free and bound states. Consistently, molecular dynamics simulations of BLIP exhibited drastic changes in the flexibility of the loop 135–145 in all complexes. Our results indicated for the first time that higher flexibility of the interdomain linker, as well as more rigidity of the interfacial loop 135–145, could be desirable determinants for enhancing inhibition of BLIP to class A β-lactamases. Together, these findings provide unique insights into the design of enhanced inhibitors.

The emergence of antibiotic resistance remains one of the most exciting challenges in the fight against bacteria, in which the β-lactam antibiotics play a central role. β-Lactamases that hydrolyze the amide bond of the β-lactam ring are primarily responsible for the inactivation of these antibiotics (1). The mechanisms underlying this process have been widely studied and several inhibitors, *e.g.*, clavulanic acid, sulbactam, tazobactam, and avibactam, are clinically used to overcome β-lactam antibiotic resistance (2). However, β-lactamases are evolving rapidly, which enables them to circumvent the inhibition provided by previously discovered compounds. The growing prevalence of bacteria producing multiple β-lactamases with enhanced hydrolytic efficiency and extended-spectrum activity is making the situation even more complicated and dangerous. Thus, novel strategies for designing inhibitors are urgently needed to tackle the problem of continuing bacterial evolution.

β-Lactamases are a large family of enzymes that can be divided into four classes (A–D), including three serine-catalyzed β-lactamase classes (classes A, C, and D) and one zinc-binding metallo-β-lactamase class (class B) (2). Class A β-lactamases are one of the most clinically prevalent types, and they pose huge community health risks. TEM (named after the patient Temoneira), SHV (a sulfhydryl variant), and PC1 (the first case of antibiotic resistance) are particularly important among the widely spread and extensively studied class A β-lactamases. These enzymes are found to possess many variants with extended spectrum of substrates and resistance to inhibitors, despite their highly conserved sequences and overall folding (3). The high homology shared by different classes of β-lactamases hints at the possibility of discovering a universal β-lactamase inhibitor.

β-Lactamase inhibitory protein (BLIP) is naturally produced by *Streptomyces clavuligerus* and consists of a tandem repeat of αβ domains conjugated by a flexible interdomain loop (Fig. 1) (4). It can bind to several types of class A β-lactamases, including *Escherichia coli* TEM1, *Klebsiella pneumoniae* SHV1, and *Staphylococcus aureus* PC1. Such property provides an alternative strategy to overcome β-lactamase-mediated antibiotic resistance, considering the recent development of various delivery systems for protein-based drugs (5). The crystal structures of BLIP/TEM1 (PDB ID: 1jtg) and BLIP/SHV1 (PDB ID: 2g2u) exhibit high structural homology, with an all-C_α_ root-mean-square-deviation (RMSD) of 0.6 Å, and the bound BLIPs show a C_α_ RMSD of 1.0 Å (Fig. S1) (6). Structural analysis suggested that the decreased volume of D104 (SHV1) compared to E104 (TEM1) removes a salt bridge with K74 (BLIP) and several contacts with the large interfacial loop. This structural information provided valuable insights, yet it did not explain the significantly different binding affinities and specificities. The binding potency of BLIP toward these β-lactamases varies, with measured K_d_ values in the subnanomolar to the micromolar range (7, 8). BLIP can bind TEM1 (K_d_ ≈ 1.3 nM) around 1000-fold tighter than SHV1 (K_d_ ≈ 1720 nM) and PC1 (K_d_ ≈ 380 nM). We speculated that other factors, *e.g.*, protein dynamics, might be critical for the binding.

**Figure 1:**
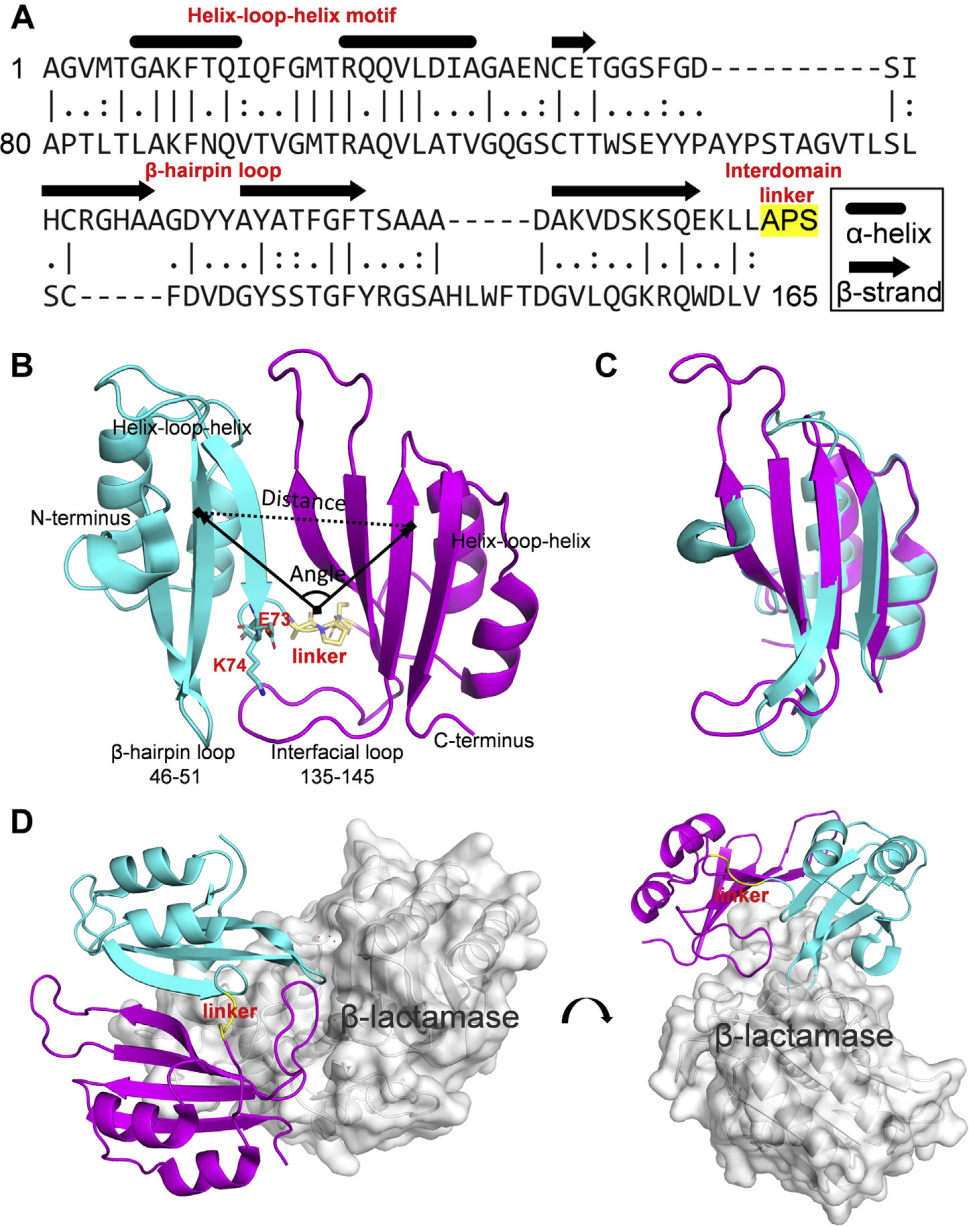
**Sequence and structure of BLIP.***A*, sequence alignment of the N-terminal (1–76) and C-terminal (80–165) domains. Secondary structures are indicated above the sequence and the three-residue linker is highlighted in *yellow*. *B*, full-length crystal structure of BLIP (PDB ID: 3gmu). The three-residue linker at the interdomain loop is labeled as *yellow sticks*. The distance and angle between the N-terminal (in *cyan*) and the C-terminal (in *magenta*) domains are indicated. *C*, the structural alignment of the N-terminal (in *cyan*) and C-terminal (in *magenta*) domains. *D*, representative crystal structures of BLIP and TEM1 β-lactamase (PDB ID: 1jtg).

Researchers have attempted to enhance the inhibitory activities of BLIP toward β-lactamases using phage display (7), computational design program (8, 9), and site-directed mutagenesis (10, 11). Reynolds *et al.* (8) redesigned the interface of BLIP and SHV1 to enhance the binding. A point mutation at E73M of BLIP improved the binding affinity by 1000-fold (K_d_ ≈ 4.4 nM) relative to the wild-type BLIP with SHV1 (K_d_ ≈ 1720 nM). Such improvement was hypothesized to be due to the restoration of a salt bridge between D104 (SHV1) and K74 (BLIP), which was present between E104 (TEM1) and K74 (BLIP). Hanes *et al.* (9) performed alanine mutagenesis on K74 (BLIP) and D104 (SHV1) based on BLIP-E73M to eliminate the salt bridge formation. BLIP-E73M/SHV1-D104A and BLIP-E73M-K74A/SHV1 showed up to 38-fold enhancement in binding affinities (with K_d_ ≈ 48 nM and 130 nM, respectively) compared with that of wild-type BLIP/SHV1. This study illustrated that the D104 (SHV1)-K74 (BLIP) salt bridge is important and that other yet undiscovered factors could enhance the binding. In this case, the elimination of the electrostatic repulsion between SHV1 residue D104 and BLIP residue E73 at the interface was one possibility. Structural alignment of BLIP/SHV1 (PDB ID: 2g2u) and BLIP-E73M/SHV1 (PDB ID: 3c4p) revealed no major changes in the overall structures of BLIP (Fig. S1) (8). Yuan *et al.* (7) have identified a potent BLIP variant, K74G, that inhibits PC1 β-lactamase with the binding affinity (K_d_ ≈ 26 nM) increased by 10-fold compared with the wild-type (K_d_ ≈ 380 nM). PC1 residue 104 is an alanine, whereas the equivalent residue is an aspartate on SHV1 and a glutamate on TEM1. PC1 A104 lacks the ability to form a salt bridge with K74 from BLIP. The mutant A104E of PC1 binds 15-fold tighter with BLIP, presumably by restoring the salt bridge between E104 and K74. However, BLIP variant K74G binds PC1 with a K_d_ ≈ 26 nM, which is significantly enhanced to a level similar to that between BLIP and PC1-A104E (K_d_ ≈ 20 nM). The underlying contribution of this mutation is still unclear. Charge matching of residue 104 (PC1) and residue 74 (BLIP) studied by the thermodynamic cycle showed that this pair of residues with either opposite charges or nonpolar sidechains could be favorable for the bindings between PC1 and BLIPs (7). Unfortunately, no crystal structure of the complex of PC1 and BLIP has been available yet.

It was proposed that the ability of BLIP to adapt to various class A β-lactamases is probably due to the flexibility between the two domains of BLIP (12). However, little work has been done on the role of flexibility or conformational dynamics of BLIP upon binding β-lactamases. Following our previous study on β-lactamases regarding their binding with BLIP (13), in this study, we focused on the conformational dynamics of BLIP and its mutants upon binding class A β-lactamases using hydrogen deuterium exchange mass spectrometry (HDX-MS) complemented with molecular dynamics (MD) simulation (14). HDX-MS can inform the hydrogen bonding and solvent accessibility of backbone amides by monitoring the exchange between the amide hydrogen and the solvent deuterium. The fundamentals and applications of this technique have been increasingly recognized in recent years (15, 16, 17, 18, 19, 20, 21). MD simulation can provide information on the atomistic motions of the protein molecule in solvent (22). Analysis of μs-ms timescale simulations can reveal the conformational dynamics under equilibrium conditions (23). MD simulation combined with HDX-MS has been demonstrated to provide complementary information about protein flexibility (24, 25, 26, 27, 28). In this study, the conformational dynamics of wild-type BLIP was investigated upon binding to β-lactamases TEM1, SHV1, and PC1. Two enhanced BLIP mutants (E73M and K74G toward SHV1 and PC1, respectively) were further investigated to explore the determinants for the improved bindings. The results have enabled us to draw a dynamic picture of BLIP at different timescales and provide unique insights into its inhibitory binding with β-lactamases.

## Results

BLIP is a potent protein inhibitor toward a variety of class A β-lactamases, and engineered BLIPs with single mutations can enhance the inhibition potency by up to 1000-fold. The protein dynamics of BLIPs in their free and bound states in complexation with three class A β-lactamases was examined by HDX-MS and MD simulation in this study. The major results from differential HDX were summarized in Table 1. Briefly, BLIP showed significant protection upon binding to TEM1 while little changes upon binding to SHV1 and PC1. For both BLIP mutant E73M and K74G, the most significant protection was observed in the interdomain region. More detailed results with respect to different regions are presented below.

**Table 1:** HDX changes of BLIP upon binding with β-Lactamases

BLIP segment	WT with TEM1	WT with SHV1	E73M with SHV1	WT with PC1	K74G with PC1
N-terminus	Protection	ND	ND	Protection	ND
10–22	ND	ND (EX1)	ND (EX1)	ND (EX1)	ND (EX1)
66–85	Protection	ND	Protection	ND	Protection
74–85	Protection	ND	Protection	ND	NA
89–101	Protection	ND	Deprotection	ND	Deprotection
137–149	Protection	ND	ND	ND	ND
143–149	Protection	ND	ND	ND	ND
C-terminus	Protection	ND	Protection	Protection	Protection

Abbreviations: NA, not applicable after the mutation; ND, no detectable change within the measurement error.

### The flexibility of interdomain region tended to be enhanced in the BLIP mutants

**Figure 2:**
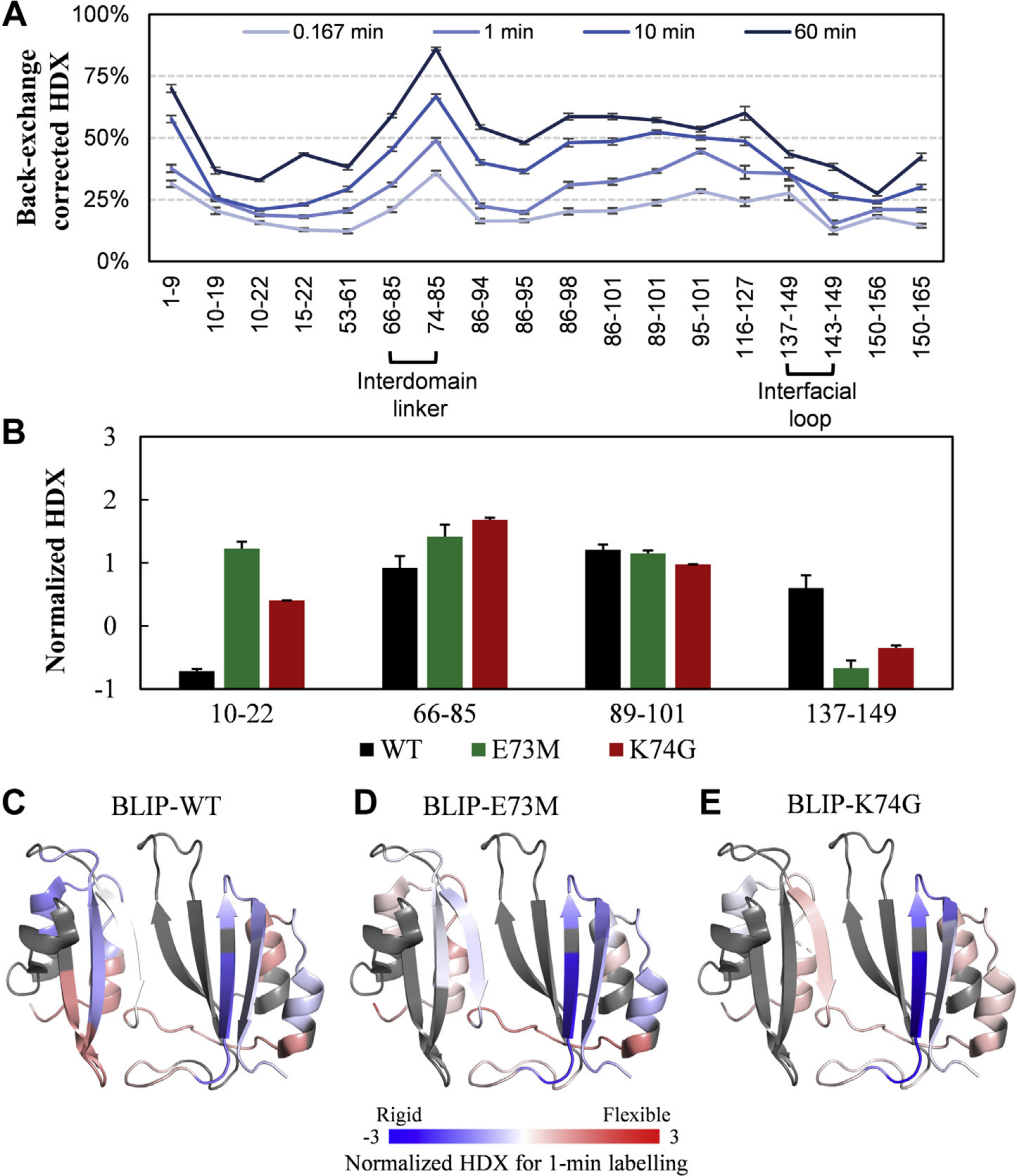
**HDX-MS profiles of unbound BLIP and its mutants.***A*, back-exchange corrected HDX for the wild-type BLIP (BLIP-WT). *B*, the normalized HDX for 1-min labeling of some key regions, including helix–loop–helix motif from the N-terminal domain (10–22), interdomain region (66–85), helix–loop–helix motif (89–101), and the β-hairpin loop (137–149) from the C-terminal domain, are compared before and after the mutation E73M (*green*) or K74G (*red*). Error bars indicate standard deviations (n = 3). HDX differences with *p*-value < 0.01 (one-tailed Student’s *t* test) were considered significant. Normalized deuterium uptakes of (*C*) BLIP-WT, (*D*) BLIP-E73M, and (*E*) BLIP-K74G for 1-min labeling are mapped onto the crystal structure of BLIP (PDB ID: 3gmu) for visualization.

**Figure 3:**
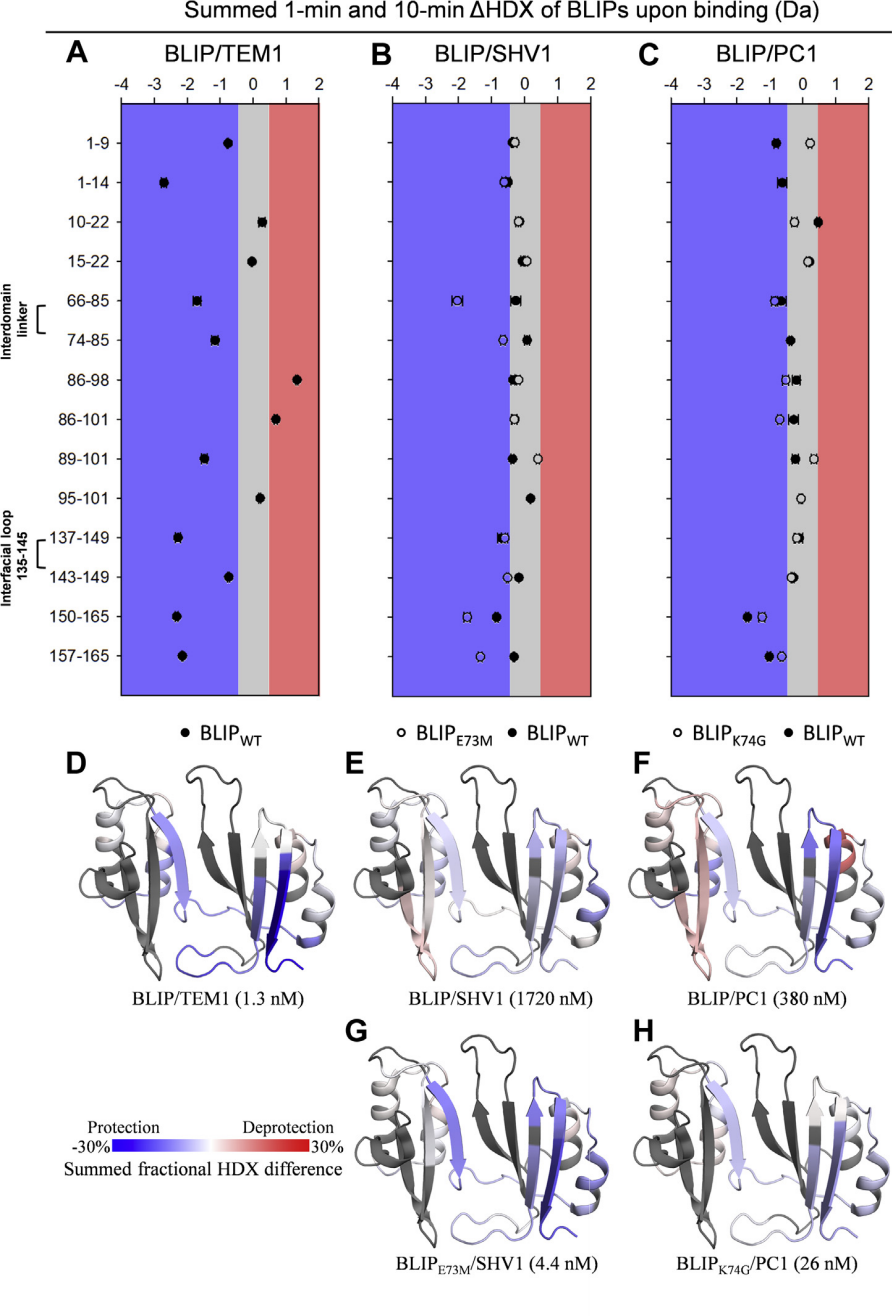
**HDX-MSresults of BLIP (*****filled circles*****) and its mutants (*****hallow circles*****) upon binding β-lactamases.***A*, binding TEM1, (*B*) binding SHV1, and (*C*) binding PC1. ΔHDX represents the difference between the free and bound states. Error bars indicate standard deviations for the time points 1 and 10 min (n ≥ 2). Positive values reflect deprotection from HDX (*red region*) while negative values reflect protection (*blue region*). Differences less than an average estimate of 95% confidence level (ca. 0.3 Da) were shown in *gray region*. HDX differences with *p*-value < 0.05 (one-tailed Student’s *t* test) were considered significant. *D–H*, the summed fractional differences in 1- and 10-min labeling are mapped onto the crystal model of BLIP (PDB ID: 3gmu) for better visualization.

BLIP has a characteristic fold with two repeating αβ domains, each of 76 residues, bridged by three interdomain residues 77–79 (Fig. 1) (5). The fragments 66–85 and 74–85 spanning the interdomain region were identified after pepsin digestion. The HDX-MS of BLIP in the free state demonstrated that the interdomain region (fragment 74–85) underwent the fastest exchange in the time course from 10 s to 60 min among all the identified fragments (Fig. 2*A*). Differential HDX-MS experiments were then performed for BLIP in the free and bound states with TEM1, SHV1, and PC1 (Fig. 3, *A*–*C*, filled circles). Our results showed that the protection of both 66–85 (1.7 Da) and 74–85 (1.2 Da) was significant upon binding to TEM1. Our observation suggested that the interdomain region was significantly less flexible upon binding to TEM1 β-lactamases. According to the crystal structure (PDB ID: 1jtg), residues 66–74 form a β-strand, which is a part of the concave of BLIP. The change in HDX upon the complex formation is more likely to be related to the rigidity conferred by the interaction interface. Interestingly, residues 75–86 form a loop conjugating the N-terminal and C-terminal domains. The HDX protection (0.5 Da) could be explained by the constrained dynamics of the interdomain movement after the binding.

**Figure 4:**
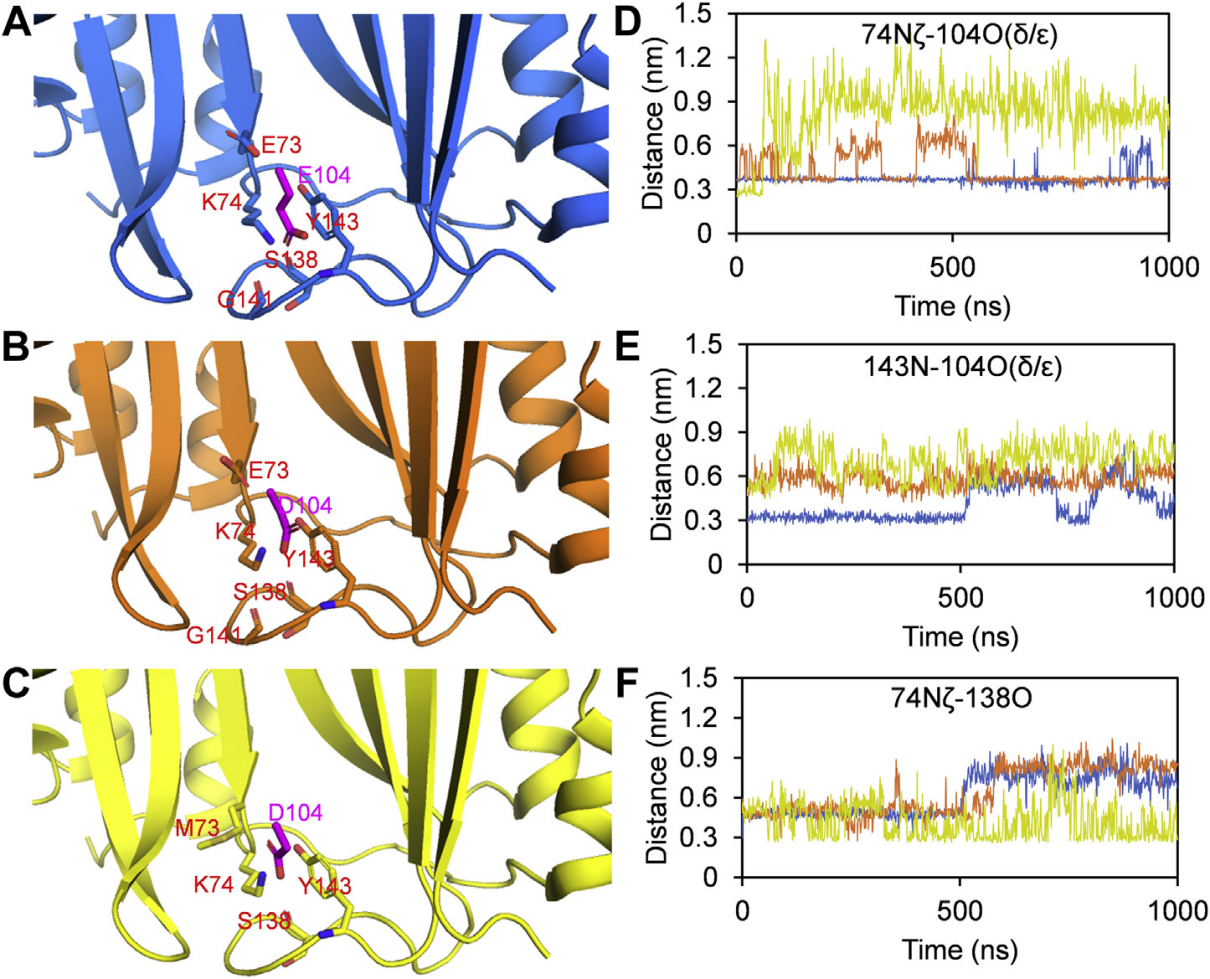
**The dynamics of the interface bonding network in the complexes.***A*, BLIP/TEM1 (PDB ID: 1jtg, *blue*), (*B*) BLIP/SHV1 (PDB ID: 2g2u, *orange*), and (*C*) BLIP-E73M/SHV1 (PDB ID: 3c4p, *yellow*) revealed by 1-μs MD simulation. Residue 104 from TEM1/SHV1 is labeled in *magenta*. The distances of hydrogen bonds for (*D*) 74–104, (*E*) 104–143, and (*F*) 74–138 were recorded from the 1-μs MD trajectories.

The flexibility of interdomain region remained unchanged upon the relatively weak bindings to SHV1 or PC1. Structural alignment of the complexes, BLIP/TEM1 (PDB ID: 1jtg) and BLIP/SHV1 (PDB ID: 2g2u), illustrated that the backbones are nearly identical in these regions (Fig. S1). Thus, it was of great interest to observe these different structural responses between these topologically similar bindings. Such variations might be due to the presence of a salt bridge between residues K74 (BLIP) and E104 (TEM1), which is absent between K74 (BLIP) and D104 (SHV1) or A104 (PC1) as previously reported (7, 8). Consistently, the 1-μs MD simulations of the complexes TEM1/BLIP and SHV1/BLIP showed that the salt bridge between D104 (SHV1) and K74 (BLIP) was less stable compared with that between E104 (TEM1) and K74 (BLIP) (Fig. 4*D*).

To investigate the roles of the interdomain region and the salt bridge, a BLIP mutant E73M, which has been reported to regain the ability to form a salt bridge between K74 (BLIP-E73M) and D104 (SHV1) (8), was examined by HDX-MS in the free state, and the results were compared with those of wild-type BLIP. The normalized HDX levels of fragments 66–85 (Figs. 2*B*) and 74–85 (Fig. S2) were significantly higher after the mutation, indicating that the BLIP E73M mutant has a more flexible interdomain linker than the wild-type BLIP. Moreover, this region (residues 66–85 and 74–85) was much more protected from HDX after binding to SHV1 (Fig. 3*B*, hallow circles). Note that fragment 74–85 actually reflected the HDX of residues 75–85 due to the fast back exchange of the N-terminal residue (K74). So, the contribution from K74 could be observed from the HDX of residues 66–85 instead of 74–85. Though the change in HDX protection was not so obvious for residues 74–85 (75–85), it was very significant for 66–85 (67–85), indicating the important contribution of residues 67–74 for the improved binding toward SHV1. Such observation might be explained by the enhanced binding interface due to the restoration of the salt bridge between K74 (BLIP-E73M) and D104 (SHV1) (6, 8). However, the 1-μs MD trajectory of the complex BLIP-E73M/SHV1 showed that the restored salt bridge was absent after 58 ns and a new intramolecular hydrogen bond between K74 and S138 was formed (Fig. 4, *D* and *F*). Thus, the reduced flexibility of the interdomain region was suggested to be contributed by either the intermolecular salt bridge between K74 (BLIP-E73M) and D104 (SHV1) or the intramolecular hydrogen bond between K74 and S138 in BLIP (Fig. 4, *D* and *F*). In either case, K74 is involved in a strong bond when BLIP-E73M is in the complexed state, which explained the increase in HDX protection of 66–85. These results reflected the dynamics of the interactions close to the binding interface, suggesting that the enhancement of the binding might be due to not only the intermolecular salt bridge but also the interplay between the interdomain region and the large interfacial loop on the BLIP side.

To further validate the role of the interdomain region, another mutation BLIP K74G that could enhance the binding between BLIP and PC1 was investigated (7). The HDX results for K74G suggested that the flexibility of fragment 66–85 was significantly increased compared with that of the wild-type (Fig. 2*B*). Fragment 74–85 was not detected presumably due to the mutation of residue 74. Differential HDX for BLIP/PC1 was performed for 1 and 10 min, and neither of these time points showed statistical differences. In contrast, HDX performed for 1, 10, and 100 min for BLIP/K74G/PC1 showed the significant protection (0.82 Da) at the 1-min labeling. Since BLIP-K74G enhances the inhibition toward PC1 only by 10-fold (*versus* almost the 1000-fold inhibition enhance of BLIP-E73M toward the wild type) and is unable to form a salt bridge between residues G74 (BLIP) and A104 (PC1), it might be reasonable to observe this small while still statistically significant change (0.82 Da, *p*-value < 0.01). Thus, the 10-fold enhancement in the binding could be due to a favorable conformational change in the interdomain region, which allows a better adaption between the binding pocket and the protruding loop from the β-lactamase.

### Cooperative effect of mutations E73M and K74G on the structural integrity of the large interfacial loop 135–145

Residues 135–145 on the C-terminal domain (CTD) form a large loop analogous to the β-hairpin (residues 46–51) on the NTD and are the most disordered region according to the B-factors observed in X-ray crystallography (PDB ID: 3gmu). Both of these topologically equivalent loops insert into the active pocket of β-lactamases (Fig. 1*D*) (12). The HDX profiles of the fragments 137–149 show significant protection from HDX (*i.e.*, enhanced integrity) upon binding with TEM1 while there were no significant changes upon binding SHV1 or PC1 (Fig. 3, *A*–*C*, filled circles). Unexpectedly, both mutants E73M and K74G on their own showed significantly reduced HDX for fragment 137–149 compared with wild-type BLIP in the free states (Fig. 2*B*). Yet, the HDX values of this large loop in E73M and K74G upon binding to SHV1 and PC1, respectively, are preserved compared with those of the BLIP mutants in their free states (Fig. 3, *B* and *C*, hollow circles). Together, these results reflected that this loop in the complexes of BLIP-E73M/SHV1 and BLIP-K74G/PC1 conferred more integrity than that in the complexes of BLIP/SHV1 and BLIP/PC1, respectively.

The K74-E104-Y143 interactive network of the complexes BLIP/TEM1 and BLIP/SHV1 differed as revealed by our MD trajectories (Fig. 4, *D* and *E*). In the case of TEM1, its E104 on the protruding loop interacts with K74 (*via* salt bridge) and Y143 (*via* hydrogen bonding) on BLIP as observed from our MD simulation (Fig. 4, *D* and *E*, blue lines), which could explain the significantly reduced flexibility of the loop 135–145 after the binding as observed by HDX-MS. In the case of SHV1, the interactive network K74-E104-Y143 is less stable than that in TEM1 during the MD simulation (Fig. 4, *D* and *E*, orange lines). Although there was no evidence that the E73M mutation on BLIP could strengthen the interactions in this network, this point mutation was shown to induce a new intramolecular hydrogen bonding between K74 and S138 after the binding (Fig. 4*F*, yellow lines). This enhanced bonding might support the change in flexibility of the loop 135–145 in the complex of SHV1/BLIP-E73M as compared with that of SHV1/BLIP. Our previous work showed that the interfacial protruding loop of β-lactamases preferred some rigidity for tight bindings with BLIP (13). Taken together, our results suggested that the integrity of the interface between the interfacial loop 135–145 of the BLIP and the protruding loop of β-lactamases could contribute to the inhibitory binding.

### N-terminal helix–loop–helix motif showed structural heterogeneity for enhanced BLIP mutants

The helix–loop–helix motifs exhibit the highest internal sequence identity and structural similarity within the repeat domains (Fig. 1, *A* and *C*). They pack against a four-stranded antiparallel β-sheet on the opposite side of the binding interface (Fig. 1*D*) (5). HDX profiles of these motifs in the NTD and CTD were analyzed upon binding the β-lactamases. The N-terminal motif spanning residues 10–22 on BLIP showed no significant differences in deuterium uptake upon binding to all tested class A β-lactamases, *i.e.*, TEM1, SHV1, and PC1, regardless of their binding affinities (Fig. 3, *A*–*C*, filled circles). In contrast, a large increase in the protection for residues 1–14 compared with 1–9 was observed in BLIP/TEM1 while not in BLIP/SHV1 or BLIP/PC1, presumably due to the conformational change for residues 10–14 (TEM1) in the N-terminal helix–loop–helix motif. More intriguingly, this region in both BLIP mutants exhibited bimodal mass distribution, which was not observed in the wild-type protein (Fig. S3) (29). Slow-exchange (blue lines) and fast-exchange (red lines) bimodal HDX reflected folded and unfolded conformations, respectively. Such HDX profiles were not significantly changed upon binding to SHV1 or PC1, indicating that the structural heterogeneity preserved in the complexes of BLIP-E73M/SHV1 and BLIP-K74G/PC1. A shorter fragment spanning residues 15–22 on both mutants also exhibited bimodal HDX profiles compared with that on the wild type (Fig. S3), further verifying the observation.

This observation was rare while particularly valuable for native proteins under physiological conditions. Our results suggested that the BLIP mutations induced an additional subpopulation of conformation with enhanced flexibility in the N-terminal helix–loop–helix motif compared with the wild type. This might lead to relaxation of this motif to facilitate a more favorable conformation upon binding to β-lactamases. Such observations might be due to the destabilizing effects of the mutations, which could alter the hydrogen deuterium exchange mechanism. For example, two mutants I106A and V108G of Ribonuclease A demonstrated strongly destabilized structures throughout the protein indicated by a shift from the EX2 to the EX1 mechanism (30). Besides, Ye *et al.* (31) observed a bimodal distribution similar to our results for peptides in the noncanonical interface, revealing the heterogeneity of the hexameric Hsp104 structure. For the first time, our results indicated that structural heterogeneity of the N-terminal helix–loop–helix motif was allosterically induced by the single mutations, *i.e.*, E73M and K74G, in the interdomain region. A more flexible conformation might be favorable for better binding with the class A β-lactamases.

The C-terminal helix–loop–helix motif (residues 89–101) of wild-type BLIP was protected from HDX upon binding to TEM1 while there were no changes in HDX upon binding to SHV1 or PC1 (Fig. 3, *A*–*C*, filled circles). Interestingly, BLIP E73M and K74G caused little changes in the flexibility of this motif compared with that of the wild type (Fig. 2*B*) and the flexibility increased upon binding to SHV1 and PC1, respectively (Fig. 3, *B* and *C*, hallow circles). These results suggested that relaxation of this helix–loop–helix motif on BLIP might provide entropic benefit to the binding toward SHV1 and PC1. Similar change was observed for the loop 135–145 in the interaction between BLIP and KPC-2 β-lactamase (32).

### The C-terminus of BLIP was protected from HDX upon the inhibitory binding

Both termini of BLIP were protected from HDX after binding to TEM1 and PC1 (Fig. 3, *A* and *C*, filled circles). By contrast, the HDX of neither terminus was significantly changed upon binding to SHV1 (Fig. 3*B*, filled circles). No significant HDX differences between the BLIP mutants and wild type were observed for either of the termini (Fig. 2, *C*–*E*). Furthermore, no significant changes were found for the N-termini of BLIP mutants after the binding (Fig. 3, *B* and *C*, hallow circles). In contrast, the C-termini of the BLIP mutants were all protected from HDX after the binding (Fig. 3, *A*–*C*, hallow circles). Our results showed that the binding with β-lactamases induced significant changes in conformational dynamics of the C-terminus rather than the N-terminus of BLIPs. The N-terminus of BLIP sits on the other side of the binding while the C-terminus is located on the edge of the concave interface (Fig. 1*D*). Moreover, the C-terminal fragments 150–165 and 157–165 in BLIP-K74G/PC1 showed decreases in protection in comparison to those of BLIP/PC1, indicating that a less rigid conformation might contribute to tighter binding in the C-terminus of the K74G mutant. In contrast, a gain in protection was observed for BLIP-E73M/SHV1 in comparison to BLIP/SHV1. Therefore, this C-terminus has a higher rigidity similar to that for the BLIP/TEM1 binding. Together, our results suggested that the deformation of the C-terminus of BLIP could lead to a better adaptation of the protruding loop from β-lactamases onto the concave interface.

### MD simulation of BLIPs revealed the fluctuation of loop regions and interdomain flexibility

**Figure 5:**
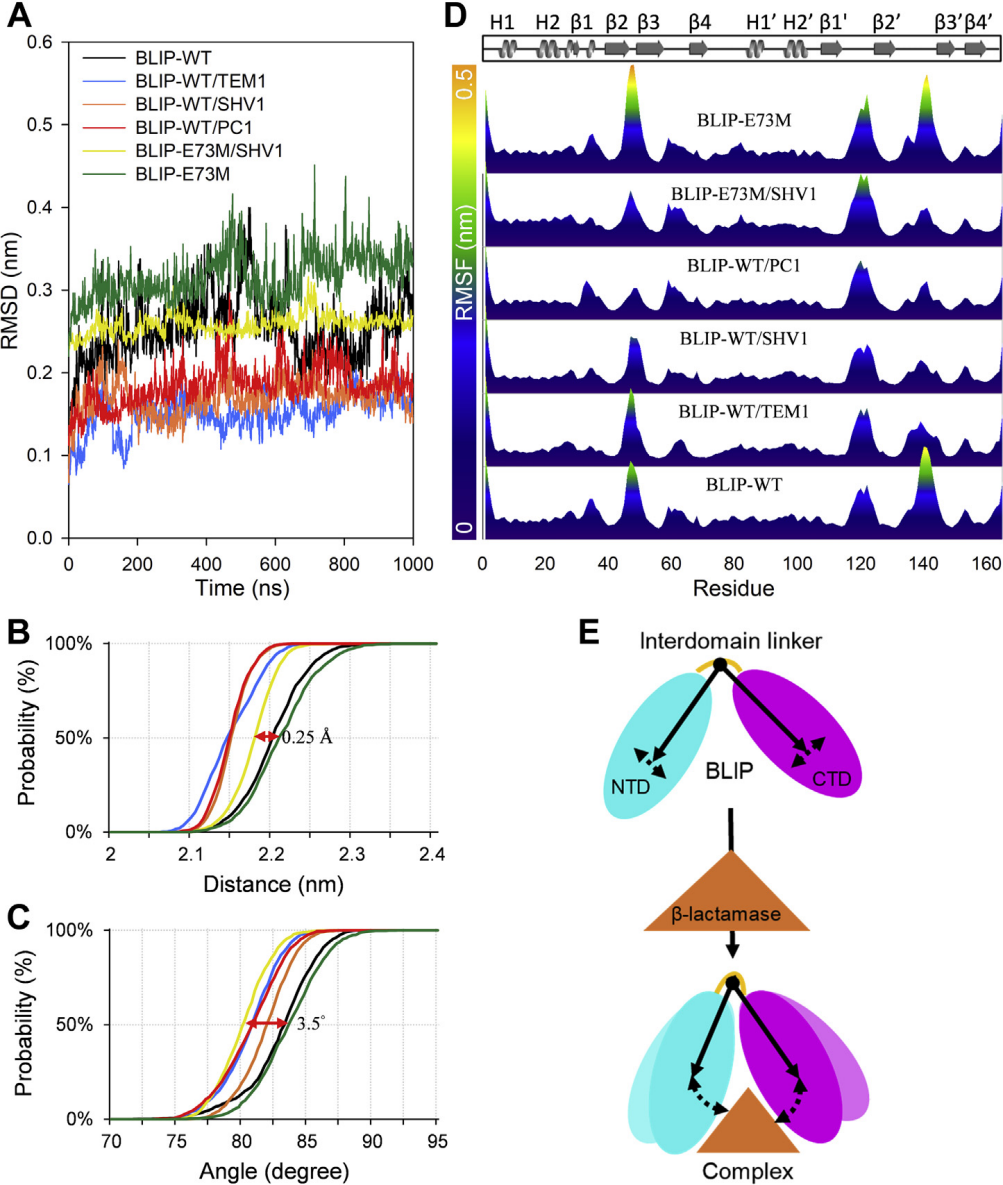
**1-μs MD simulation of BLIPs and their complexes with β-lactamases.***A*, the RMSD of BLIP in different states during 1-μs MD simulation. *B* and *C*, probability plots of the distance and angle between the N-terminal and the C-terminal domains of BLIPs in its free state and bound state from the 1-μs MD trajectories. The *arrows* indicate the change in the distance and angle for BLIP-E73M upon binding SHV1. *D*, the RMSF of BLIP in in different states was plotted per residue from the 1-μs MD trajectories. *E*, schematic presentation indicates inhibitory binding between BLIP and β-lactamases contributed by the interdomain flexibility.

MD simulation can provide explicit information on the dynamics of BLIPs, which is complementary to the HDX-MS results. 1-μs MD simulations of BLIP were performed in its free and bound states with TEM1, SHV1, and PC1 (Fig. 5*A*). When the protein backbones were compared, the most obvious change was located at the large loop consisting of residues 135–145 where the fluctuation was significantly decreased for all bound states (Fig. 5*D*). This was in good agreement with the HDX-MS results, which indicated that this loop, as a major binding interface, would become more rigid upon the tight binding to the β-lactamases. The analogous loop 46–51 on the NTD that was not covered in the HDX-MS study underwent changes to varying extents upon binding to different β-lactamases. The binding to SHV1 induced a moderate change in flexibility while the binding to PC1 caused the most significant increase in rigidity at this region. By contrast, this loop showed little change in the complex with TEM1. These results complemented the uncovered part in the HDX-MS study.

BLIP mutant E73M on its own was simulated in comparison with the wild-type BLIP (Fig. 5*A*). The mutant E73M showed increased fluctuation (maximum at residue T82) at the interdomain region next to the mutation site (Fig. S4). It is interesting to see that mutation E73M has little effects on itself but notable effects on its N-terminal and C-terminal residues. This observation agreed with our HDX-MS results that showed improved flexibility in the interdomain region. Besides, significantly enhanced flexibility was found at the β-hairpin 46–51 (Fig. 5*D*), which is known to be the major active site (12). Furthermore, a loop conjugating β1′ and β2′ (residues 115–125) that is distant from the interface was found more flexible in the mutant E73M and remained flexible upon binding to SHV1. Together, these results indicated that the affinity of BLIP E73M toward SHV1 could be enhanced *via* destabilized loop regions from both NTD and CTD, which showed enhanced interdomain flexibility (33).

To further investigate the interdomain flexibility of BLIP, the distance and angle between the NTD and CTD were measured during the 1-μs MD simulation (Fig. 5, *B* and *C*) (29). The distance is defined by the centers of mass (COM) of NTD and CTD and the angle is defined by two vectors, from the COM of the three-residue (Ala77-Ser79) interdomain linker to the COMs of the NTD and the CTD, respectively (Fig. 1*B*). The distance and angle were extracted from the MD trajectories and plotted against the probability. The plots showed S-shaped probability curves due to the normal distribution of the MD ensembles. The distance was reduced by 0.5 Å upon binding to all the class A β-lactamases under investigation (Fig. 5*B*). The angle was decreased by 2.5° upon binding to TEM1 and PC1 while half decrement was observed upon binding to SHV1 (Fig. 5*C*). This finding is consistent with the conformational change in the crystal structure where a 5.3° inward rotation of the two analogous domains was observed (12).

The BLIP mutant E73M exhibited little changes in the orientation between NTD and CTD when compared with the wild type (Fig. 5, *B* and *C*, black *versus* green lines). Interestingly, the distance decreased by 0.25 Å while the angle decreased by 3.5° upon binding to SHV1 (Fig. 5, *B* and *C*, yellow *versus* orange lines). Such changes might support a more favored inward rotation of the NTD and CTD for BLIP-E73M upon binding to SHV1. These results agreed well with our HDX-MS results, indicating that the affinity of BLIP toward these class A β-lactamases might be modulated by the interdomain orientation, *i.e.*, a structural feature that relates to the flexibility of the interdomain region. A more flexible interdomain linker on BLIP could result in a more dynamic pocket that could adapt better with SHV1 (Fig. 5*E*). Thus, the modulation of the interdomain orientation could be critical in the enhancement of the binding affinity and specificity of BLIP toward class A β-lactamases.

## Discussion

The abilities to widely adapt to several class A β-lactamases might be contributed by the flexibility conferred by the two-domain architecture of BLIP, but no solid evidence has been reported yet (12). In this study, compared with the wild-type BLIP, both BLIP mutants (E73M and K74G) were significantly more flexible in the interdomain region, where significant protection from HDX was observed upon binding to SHV1 and PC1, respectively. The MD simulation showed the presence of an intramolecular salt bridge between E73 and K74 in the wild-type BLIP upon binding with the three β-lactamases, which might affect the flexibility of the interdomain region (Fig. S5). However, BLIP mutants E73M and K74G are unable to form this salt bridge and, therefore they have enhanced flexibility of the interdomain region, as revealed by our HDX-MS results. Our results indicated that the conformational dynamics of the interdomain region for the E73M and K74G mutants could be important to improve the inhibitory binding toward class A β-lactamases, suggesting that the relative orientation of the two domains of BLIP could be impacted by the flexibility of the interdomain linker. Such interdomain dynamics allowed BLIP to adapt onto a concave interface to bind a variety of β-lactamases.

Notably, E73 and K74 also interact with the residues in the loop 135–145 (Fig. S5), suggesting the correlation between the interdomain region and the large interfacial loop. This supported our observation that the single mutation on the interdomain region would cooperatively alter the conformational dynamics of the large interfacial loop. Furthermore, the BLIP mutant E73M-F142A would lose high affinity to SHV1, resulting in a K_d_ value that is similar to that of wild-type BLIP (9). Such evidence could support the notion that the cooperativity between the interdomain region and the large interfacial loop was essential for the enhanced inhibitory binding. More integrity of the large interfacial loop 135–145 in the E73M and K74G mutants in comparison with that of the wild type could be favorable for their enhanced bindings with SHV1 and PC1, respectively.

The conformational dynamics of the interdomain linker of multidomain proteins has been proven to be able to modulate the orientation of the domains, a feature that can be critical for ligand binding (34) and functioning of chaperon protein (35). For instance, the orientation of the PDZ domains connected by a conserved peptide linker facilitates the binding to multiple targets and the binding affinity can be modulated by the interdomain rearrangements (34). A recent study on a chaperon protein SurA using HDX-MS also revealed the important role of the interdomain dynamics in multiple binding to protein clients (35). In this study, by using HDX-MS and site-directed mutagenesis, for the first time, we revealed that the increased flexibility of the interdomain region in BLIP could allow better adaption of the β-lactamases into the binding pocket of BLIP.

Overall, novel insights into the mechanism underlying the broad and enhanced binding of BLIP mutants toward various β-lactamases were presented in this study. These findings revealed the role of the inhibitor protein from the perspective of protein dynamics. Engineered BLIPs with enhanced potency exhibited critical changes in the plasticity of interfacial loops and interdomain orientation. Such insights into the conformational dynamics of BLIPs can provide general guidelines toward the designing of novel inhibitors.

## Experimental procedures

### Hydrogen deuterium exchange mass spectrometry

Protein expression and purification were described as previously reported (13). For typical HDX-MS experiments, 20 μM BLIP or BLIP mutant was incubated with various β-lactamases at a molar ratio, which ensures more than 90% occupancy of BLIP (see details in Tables S1–S3). Hydrogen deuterium exchange was initiated by diluting 3 μl protein sample into 27 μl D_2_O (99.9%, Cambridge Isotope Laboratories) buffer (100 mM phosphate buffer in 90% D_2_O, pD 7.4). The proteins were labeled for various time intervals for local HDX (optimized from global exchange for 10 s, 1 min, 10 min, and 60 min, as shown in Fig. S6, and extended to 100 min to confirm the bimodal distribution of peptide 10–22 in BLIP mutants E73M and K74G). The reaction was quenched, followed by on-line pepsin digestion (passed by for global exchange), and analyzed by LC-MS as described previously (13). The coverage of BLIP spanned from 48% to 64%, with the uncovered region mainly involving two pairs of disulfide bonds (Cys30-Cys42 and Cys109-Cys131). Optimization of the digestion condition was implemented, *i.e.*, adding 100–500 mM TCEP to the quenching buffer (36), but little improvement was achieved, indicating the difficulty for the disulfide bonds to be reduced in the short time (*i.e.*, 3–5 min under the quenching condition). Fortunately, the uncovered region did not include the interdomain region and the major binding interface, thus showing little effects on our major conclusions. More experimental details are provided in Tables S1–S3.

Back-exchange rate (BE%) was estimated to be an average of 40% using wild-type BLIP, with detailed back exchange for each peptide shown in Figure S7. Briefly, the peptides were collected after online pepsin digestion and the solvent was removed before D_2_O buffer was added for labeling for at least 4 h to allow maximum deuteration. The fully labeled peptides were analyzed by a typical HDX experiment. The back-exchange rate was calculated according to Equation 1.

(1) $$BE\%=1-\frac{HDX_{peptide}}{Exchangable\;NH}\times 100\%$$

The local HDX was only back-exchange corrected for the wild-type BLIP to see the deuteration across the protein sequence (Equation 2) and was not corrected when doing comparison in free and bound states.

(2) $$HDX_{BE\;corrected\;}=\frac{HDX_{observed}}{BE\%}$$

The fractional deuterium uptake was calculated as reference to the maximum exchangeable amide hydrogens (Equation 3).

(3) $$Fractional\;HDX=\frac{HDX_{observed}}{Exchangable\;NH}\;\times 100\%\;$$

The deuterium uptake of BLIP wild type and mutants was normalized to allow semiquantitative comparison of the results obtained in different HDX runs (Equation 4).

(4) $$Normalized\;HDX=\frac{HDX_{observed}-\mu_{HDX}}{\sigma_{HDX}}$$

where $\mu_{HDX}$ is the mean and $\sigma_{HDX}$ is the standard deviation of all observed HDX for peptides in the same HDX run. The normalization was used to compensate the variations of the HDX experiments performed on different days and allow better comparison of the results from different HDX runs. Differences in deuterium uptake with 95% confidence level (*p*-value < 0.05) were considered as a significant change for differential HDX. Uptake profiles were plotted by SigmaPlot (Systat Software Inc). The relative fractional deuterium uptake was mapped onto the crystal model of proteins using PyMol 2.0.7 (Schrodinger, LLC). Bimodal mass spectra were analyzed by HX-Express2 (32).

### Molecular dynamics simulation

The structure of wild-type BLIP was obtained from protein data bank (PDB ID: 3gmu). The structure for BLIP mutant E73M was obtained from the crystal structure of the protein complex BLIP-E73M/SHV1 (PDB ID: 3c4p). MD simulation of BLIP and its complexes with TEM1, SHV1, and PC1 was modified from the procedures that were previously described (13), also followed by BLIP mutant E73M and its complex with SHV1. 1 μs of unrestrained MD simulations were performed using GROMACS 2019 with the Charmm27 all-atom force field (37). The time step was 2 fs and configurations were saved every 1 ns. All trajectories were subjected to evaluation by calculating the root mean square deviation (RMSD) of backbone positions referenced to the crystal structure of wild-type BLIP (PDB ID: 3gmu) and further analyzed by calculating their root mean square fluctuation (RMSF) of backbone positions. The distance between NTD (1–76) and CTD (80–165) was defined by the center of mass of individual domains. The angle was defined by two vectors pointing from the center of mass of the three-residue linker (77–79) to that of individual domain. The distance and angle were sampled along the whole trajectory and probability was plotted against the distance and angle. The hydrogen bonds (with the donor–acceptor distance cutoff at 0.3 nm) and salt bridges (with the oxygen–nitrogen distance cutoff at 0.32 nm) were analyzed by VMD (version 1.9.3) (38).

## Data availability

All data are contained within the article and accompanying supporting information.

## Supporting information

This article contains supporting information.

## Conflict of interest

The authors declare that they have no conflicts of interest with the contents of this article.

## Supporting information

Tables S1–S3 and Figures S1–S7

Source data file for Figs. S1, S2, 2A, and 3
