# Supplementary material for: Interdomain flexibility and interfacial integrity of β-lactamase inhibitory protein (BLIP) modulate its binding to class A β-lactamases
Source: J Biol Chem. 2021 Jul 21;297(2):100980. doi: 10.1016/j.jbc.2021.100980 (PMC8363833; doi:10.1016/j.jbc.2021.100980)
Supplement: Tables S1–S3 and Figures S1–S7 [file mmc1.pdf]

Supporting Information

# Interdomain Flexibility and Interfacial Integrity of β-Lactamase Inhibitory Protein (BLIP) Modulate Its Binding to Class A β-Lactamases

Liwen Huang,[1,2] Pui-Kin So,[1] Yu Wai Chen,[1] Yun-Chung Leung,[1] Zhong-Ping Yao[1,2*]

[1]State Key Laboratory of Chemical Biology and Drug Discovery, Research Institute for Future Food and Department of Applied Biology and Chemical Technology, The Hong Kong Polytechnic University, Hung Hom, Kowloon, Hong Kong Special Administrative Region, China

[2]State Key Laboratory of Chinese Medicine and Molecular Pharmacology (Incubation) and Shenzhen Key Laboratory of Food Biological Safety Control, Shenzhen Research Institute of The Hong Kong Polytechnic University, Shenzhen 518057, China

***Corresponding Author**

Zhong-Ping Yao

Department of Applied Biology and Chemical Technology

The Hong Kong Polytechnic University

Hung Hom, Kowloon

Hong Kong

Tel: +85234008792

Email: zhongping.yao@polyu.edu.hk

**Contents:**

Tables S1-S3

Figures S1-S7

**Table S1.** HDX summary for BLIPs in free states

| Data Set | Free BLIP-WT | Free BLIP-E73M | Free BLIP-K74G |
|---|---|---|---|
| HDX reaction details | 100 mM Phosphate buffer in 90% $D_2O$, pH 7.4, room temperature | 100 mM Phosphate buffer in 90% $D_2O$, pH 7.4, room temperature | 100 mM Phosphate buffer in 90% $D_2O$, pH 7.4, room temperature |
| HDX time (min) | 0.17, 1, 10, 60 | 1,10,100 | 1,10,100 |
| Number of peptides | 17 | 19 | 16 |
| Sequence coverage | 48% | 58% | 57% |
| Average peptide length / Redundancy | 10 / 2.34 | 9.2 / 2.23 | 10.5 / 1.91 |
| Replicates (technical) | 3 | 3 | 3 |
| Repeatability | 0.040 Da | 0.057 Da | 0.038 Da |
| Significant differences in HDX | 0.16 Da | 0.21 Da | 0.15 Da |

**Table S2.** HDX summary for wild-type BLIP bound with β-lactamases

| Data Set | BLIP-WT bound with TEM1 | BLIP-WT bound with SHV1 | BLIP-WT bound with PC1 |
|---|---|---|---|
| HDX reaction details | 100 mM Phosphate buffer in 90% $D_2O$, pH 7.4, room temperature | 100 mM Phosphate buffer in 50% $D_2O$, pH 7.4, room temperature | 100 mM Phosphate buffer in 50% $D_2O$, pH 7.4, room temperature |
| Concentration of enzyme/inhibitor (μM) | 10/10 | 44/10 | 13.2/6 |
| Dissociation constant (nM) | 1.3[a] | 1720[a] | 380[b] |
| HDX time (min) | 1, 10 | 1, 10 | 1, 10 |
| Number of peptides | 17 | 18 | 17 |
| Sequence coverage | 48% | 64% | 64% |
| Average peptide length / Redundancy | 10 / 2.34 | 11 / 1.99 | 11 / 1.91 |
| Replicates (technical) | 3 | 2 | 2 |
| Repeatability | 0.055 Da | 0.035 Da | 0.070 Da |
| Significant differences in HDX | 0.19 Da | 0.22 Da | 0.29 Da |

[a]Obtained from: K.A. Reynolds, M.S. Hanes, J.M. Thomson, A.J. Antczak, J.M. Berger, R.A. Bonomo, J.F. Kirsch, T.M. Handel, Computational redesign of the SHV-1 β-lactamase/β-lactamase inhibitor protein interface. *J. Mol. Biol.,* **382,** 2008, 1265-1275.

[b]Obtained from: J. Yuan, D.-C. Chow, W. Huang, T. Palzkill, Identification of a β-lactamase inhibitory protein variant that is a potent inhibitor of Staphylococcus PC1 β-lactamase. *J. Mol. Biol.,* **406,** 2011, 730-744.

**Table S3.** HDX summary for BLIP mutants bound with β-lactamases

| **Data Set** | **BLIP-E73M bound with SHV1** | **BLIP-K74G bound with PC1** |
|---|---|---|
| HDX reaction details | 100 mM Phosphate buffer in 90% $D_2O$, pH 7.4, room temperature | 100 mM Phosphate buffer in 90% $D_2O$, pH 7.4, room temperature |
| Concentration of enzyme/inhibitor (μM) | 20/20 | 20/20 |
| Dissociation constant (nM) | 4.4[a] | 26[b] |
| HDX time (min) | 1, 10, 100 | 1, 10, 100 |
| Number of peptides | 19 | 16 |
| Sequence coverage | 58% | 57% |
| Average peptide length / Redundancy | 9.2 / 2.23 | 10.5 / 1.91 |
| Replicates (technical) | 3 | 3 |
| Repeatability | 0.067 Da | 0.070 Da |
| Significant differences in HDX | 0.22 Da | 0.21 Da |

[a]Obtained from: K.A. Reynolds, M.S. Hanes, J.M. Thomson, A.J. Antczak, J.M. Berger, R.A. Bonomo, J.F. Kirsch, T.M. Handel, Computational redesign of the SHV-1 β-lactamase/β-lactamase inhibitor protein interface. *J. Mol. Biol.*, **382,** 2008, 1265-1275.

[b]Obtained from: J. Yuan, D.-C. Chow, W. Huang, T. Palzkill, Identification of a β-lactamase inhibitory protein variant that is a potent inhibitor of Staphylococcus PC1 β-lactamase. *J. Mol. Biol.*, **406,** 2011, 730-744.

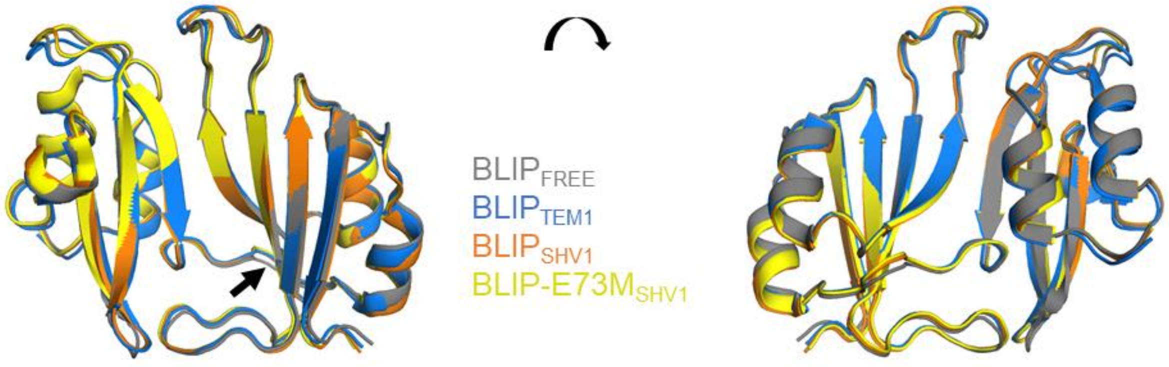


**Fig. S1.** Structural alignment of BLIPs in the free state (PDB ID: 3gmu, grey) with those in the complexes of BLIP/TEM1 (PDB ID: 1jtg, blue), BLIP/SHV1 (PDB ID: 2g2u, orange) and BLIP-E73M/SHV1 (PDB ID: 3c4p, yellow). The arrows point to the interdomain region.

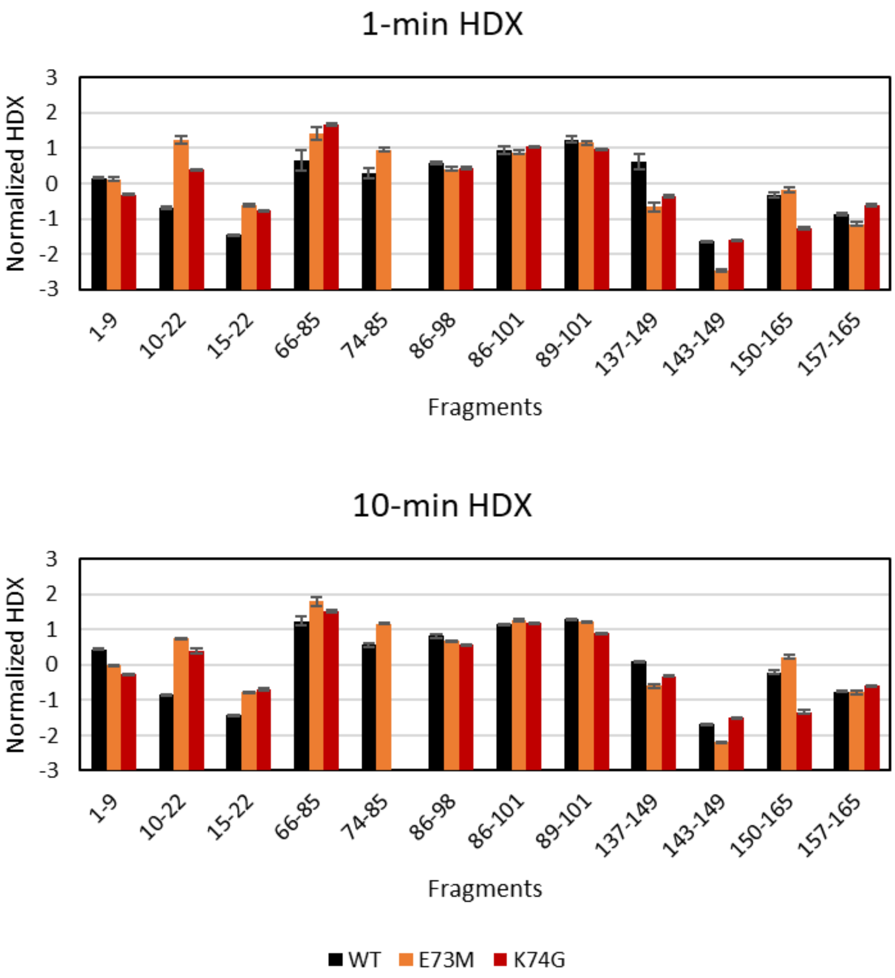


**Fig. S2.** HDX-MS profiles of unbound BLIP and its mutants E73M and K74G. Error bars indicate standard deviations for the time points 1 and 10 min (n = 3).

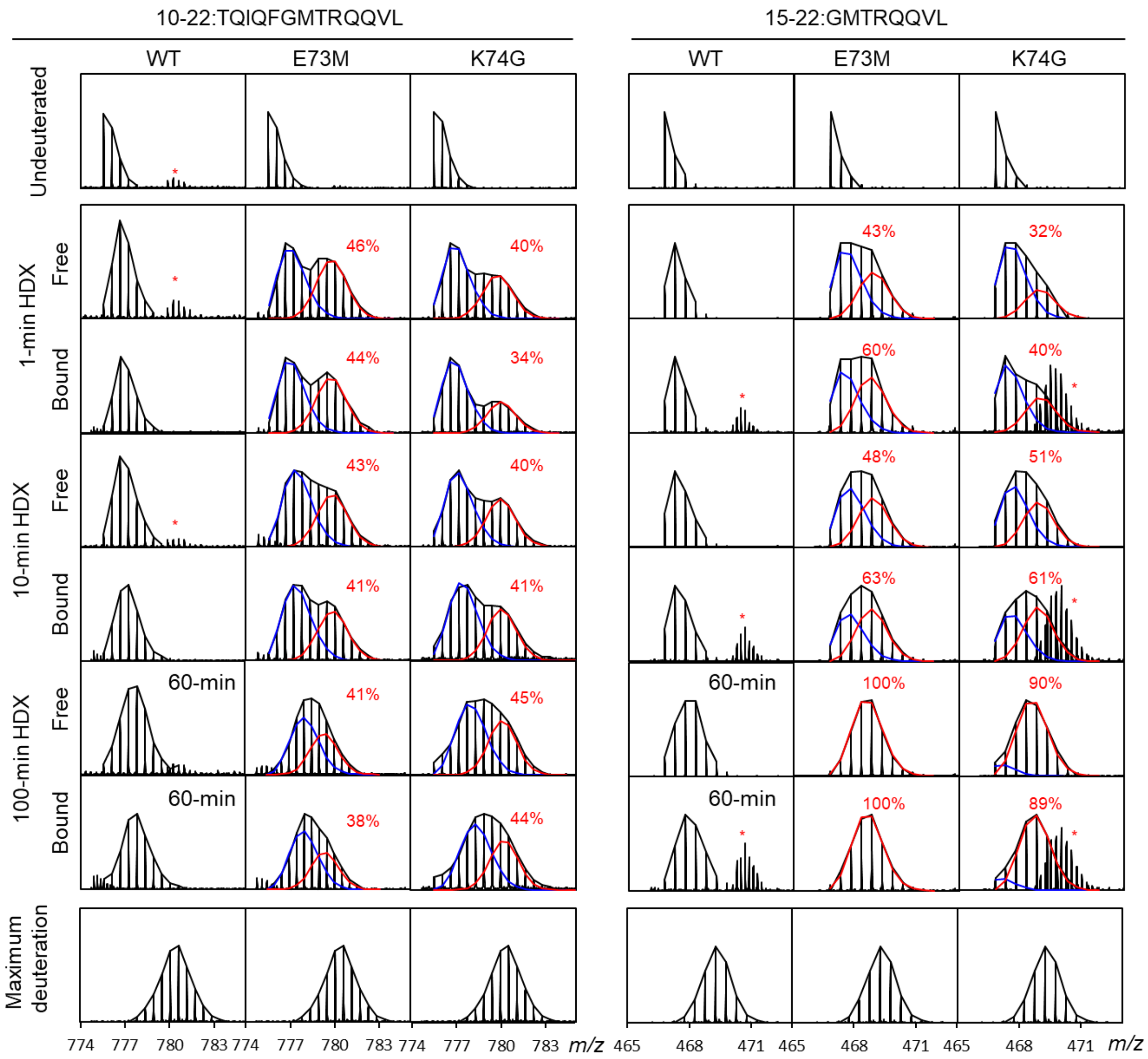


**Fig. S3.** Bimodal HDX of the N-terminal helix-loop-helix motif spanning 10-22 and 15-22 on the BLIP mutants E73M and K74G upon binding SHV1 and PC1, respectively. Representative mass spectra are shown for this motif in the free and bound states. Black lines indicate mass envelopes. The low-mass (slow-exchange and protected) and high-mass (fast-exchange and unprotected) binomial distributions are well-fitted to the mass envelops and are shown in blue and red, respectively. Fraction of the unprotected is annotated above the corresponding peak. Asterisks indicate mass envelops of other peptides.

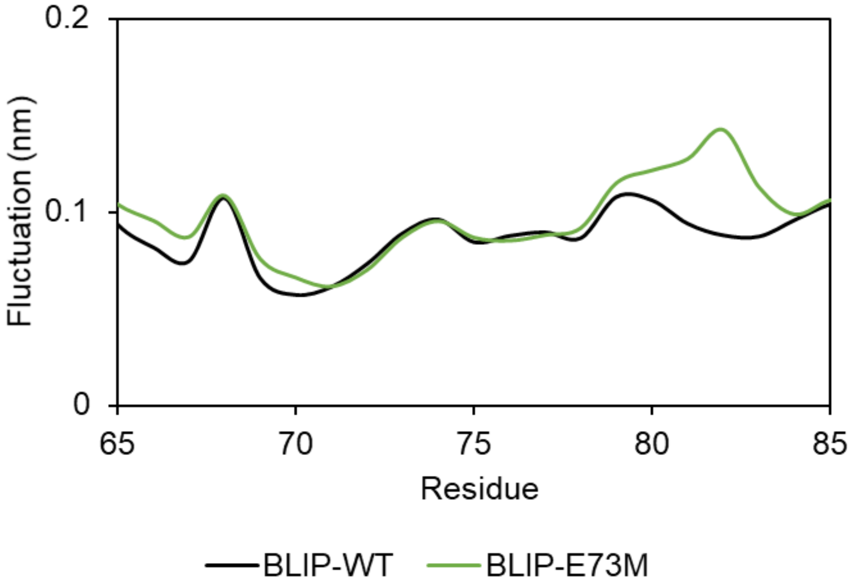


**Fig. S4.** The fluctuation of interdomain region in the wild type and E73M mutant of BLIP.

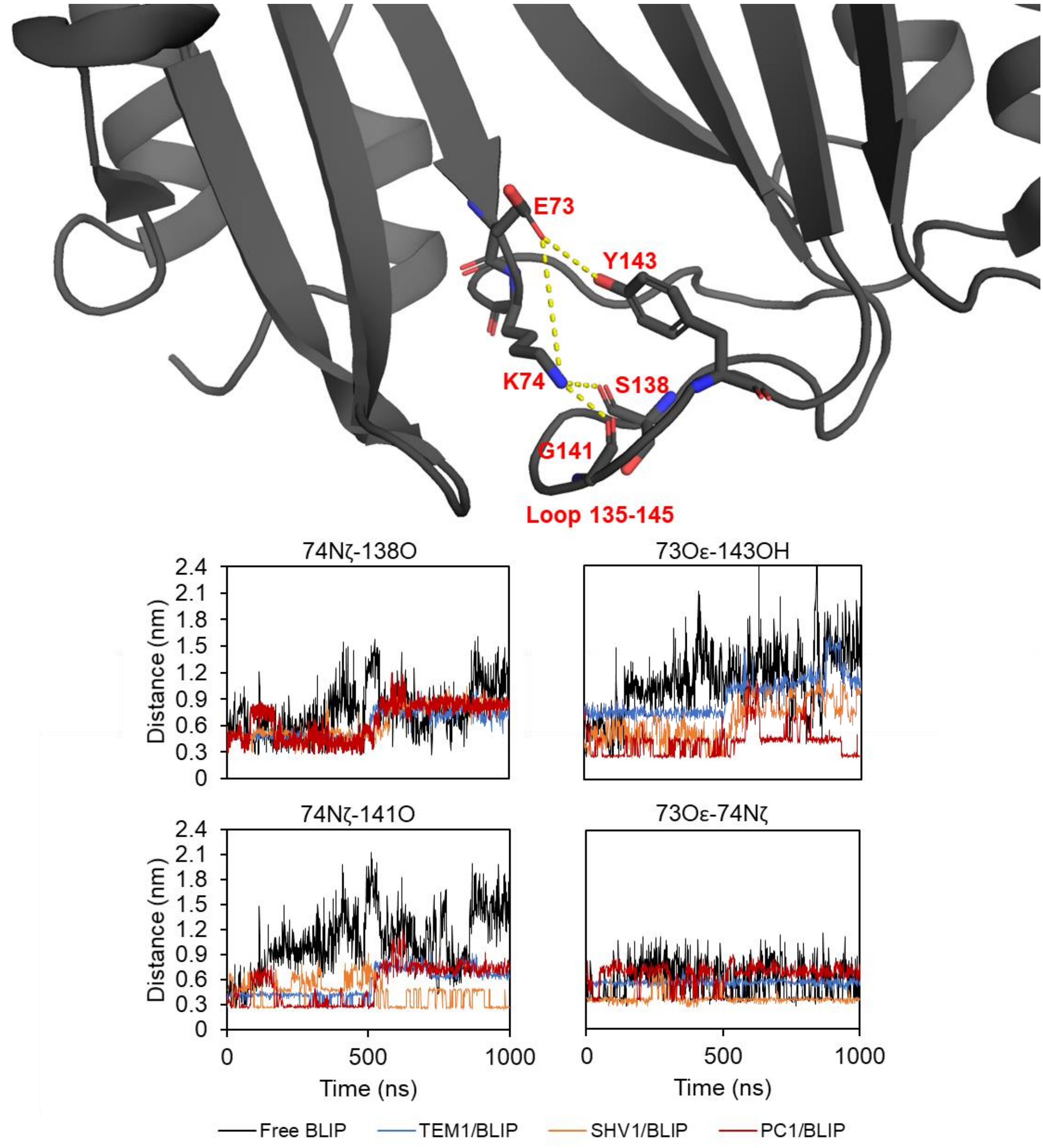


**Fig. S5.** Hydrogen bonding network in BLIP upon binding with β-lactamases (PDB ID: 3gmu). E73 and K74 interact loop 135-146 via hydrogen bond with Y143, G141 and S138.

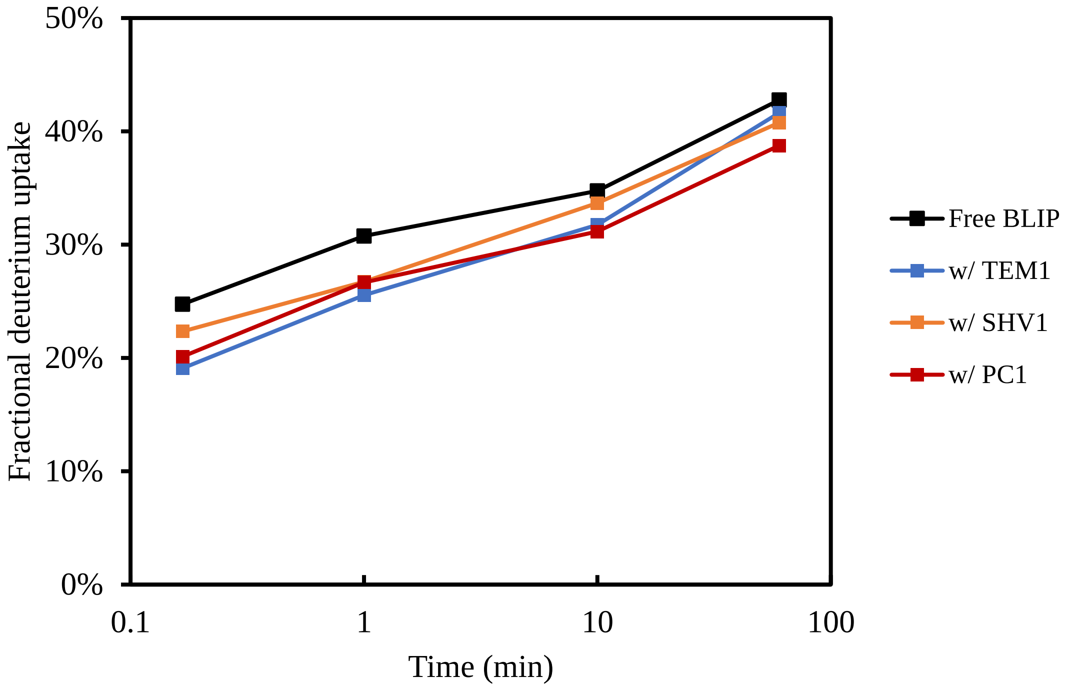


**Fig. S6.** Deuterium uptake curves for global HDX (10 sec, 1 min, 10 min and 60 min) of BLIP in free and bound states with TEM1, SHV1 and PC1.

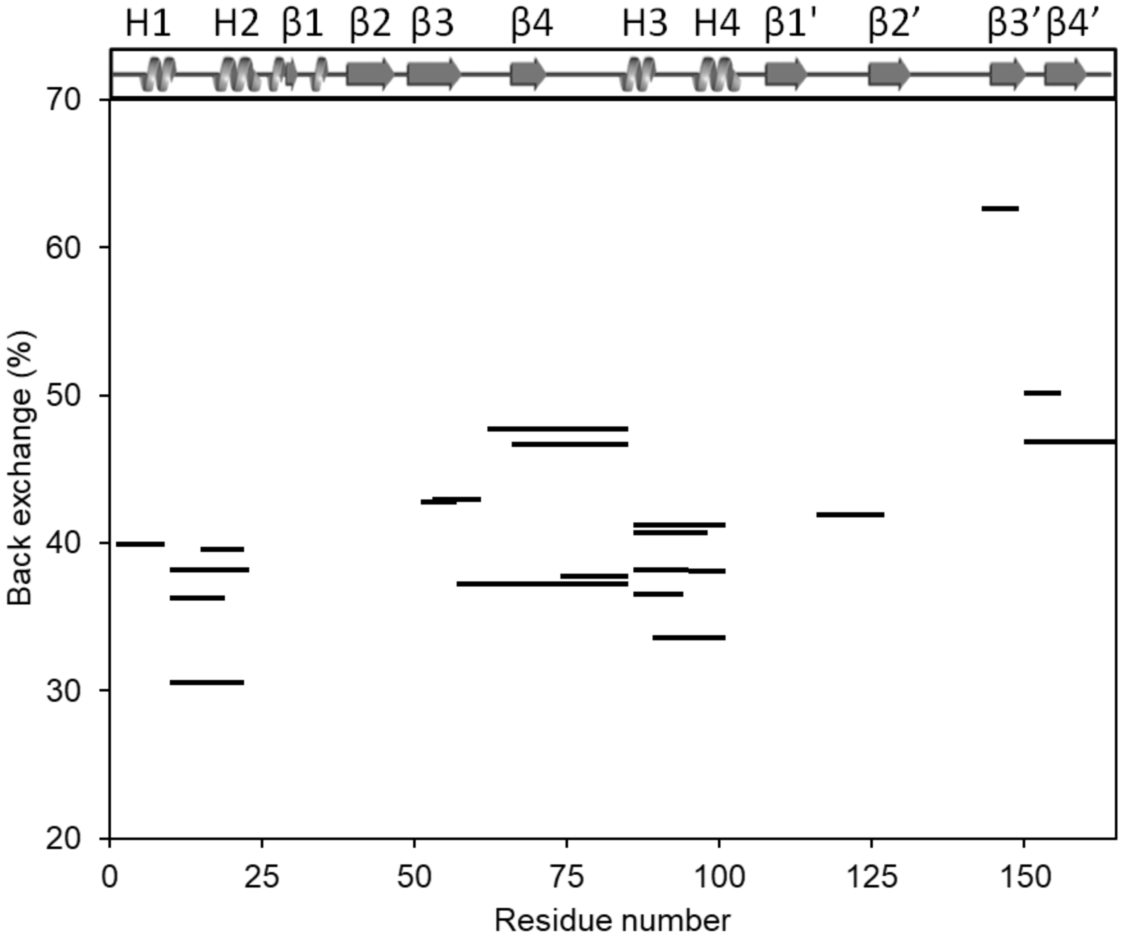


**Fig. S7.** Back exchange and coverage map of BLIP. Identified peptides were mapped on the wood plot of back exchange rate versus residue number. The average length of the peptides was around 11. The average back exchange rate was 40%. 48-64% of the amino acid sequence of BLIP was covered for HDX-MS analysis.
